# Case report: Left bundle branch pacing guided by real-time monitoring of current of injury and electrocardiography

**DOI:** 10.3389/fcvm.2022.1025620

**Published:** 2022-11-09

**Authors:** Jiabo Shen, Longfu Jiang, Hao Wu, Hengdong Li, Jinyan Zhong, Lifang Pan

**Affiliations:** ^1^Department of Cardiology, Hwa Mei Hospital, University of Chinese Academy of Sciences, Ningbo, China; ^2^Department of Global Health, Ningbo Institute of Life and Health Industry, University of Chinese Academy of Sciences, Ningbo, China

**Keywords:** left bundle branch pacing, current of injury (COI), electrocardiogram (ECG), intracardiac electrogram, continuous recording technique

## Abstract

**Background:**

Left bundle branch (LBB) pacing (LBBP) has recently emerged as a physiological pacing mode. Current of injury (COI) can be used as the basis for electrode fixation position and detection of perforation. However, because the intermittent pacing method cannot monitor the changes in COI in real time, it cannot obtain information about the entire COI change process during implantation.

**Case summary:**

Left bundle branch pacing was achieved for treatment of atrioventricular block in a 76-year-old female. Uninterrupted electrocardiogram and electrogram were recorded on an electrophysiology system. In contrast to the interrupted pacing method, this continuous pacing and recording technique enables real-time monitoring of the change in ventricular COI and the paced QRS complex as the lead advances into the interventricular septum. During the entire screw-in process, the COI amplitude increased and then decreased gradually after reaching the peak, followed by a small but significant, rather than dramatic, decrease.

**Conclusion:**

This case report aims to demonstrate the clinical significance of changes in COI and QRS morphology for LBBP using real-time electrocardiographic monitoring and filtered and unfiltered electrograms when the lead is deployed using a continuous pacing technique. The technique could be used to confirm LBB capture and avoid perforation.

## Introduction

Left bundle branch (LBB) pacing (LBBP) provides stable pacing parameters by directly capturing the conduction system in the left ventricular sub-endocardium (1). The pacing lead should be deployed deep enough into the ventricular septum to capture LBB; however, this carries the risk of perforation during lead implantation. A decrease in unipolar impedance, loss of capture, and absence of current of injury (COI) are signs of septal perforation (2). Previous studies used the intermittent pacing method to interruptedly monitor COI and paced QRS morphology, which may miss a lot of information during the transseptal implantation and enable observation of real-time changes in COI only when the lead is retracted (3, 4). One such study demonstrated that the real-time COI value during screwing in should be widely adopted in daily practice (4). Our study aimed to obtain continuous parameters from real-time monitoring of the surface electrocardiogram (ECG) and filtered and unfiltered intracardiac electrogram (EGM) to guide lead deployment.

## Case report

**FIGURE 1:**
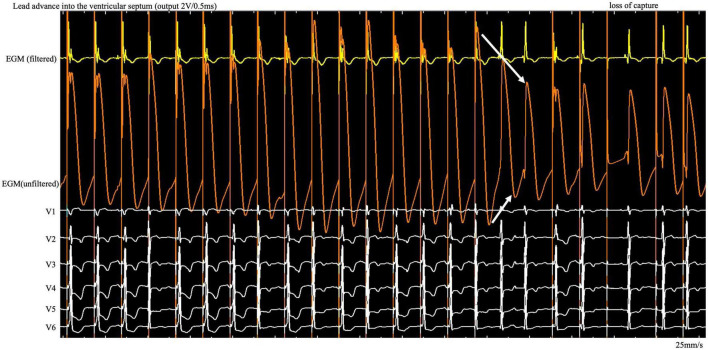
During the entire screw-in process, the COI amplitude increased and then decreased gradually after reaching the peak, followed by a small but significant abrupt decrease. COI, current of injury; ECG, electrocardiogram; EGM, intracardiac electrogram.

**FIGURE 2:**
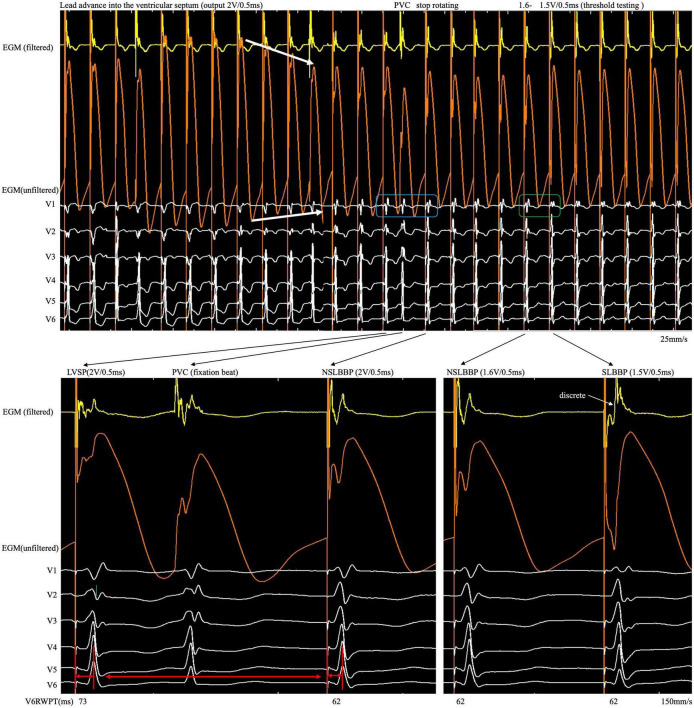
After the COI amplitude increased to its highest value, the electrode was rotated very slowly to avoid a sudden drop in COI until the LBB area was reached. An abrupt shortening of V6RWPT, discrete EGM and fixation beat were observed. COI, current of injury; LBB, left bundle branch; LBBP, left bundle branch pacing; SLBBP, selective left bundle branch pacing; NSLBBP, non-selective left bundle branch pacing; ECG, electrocardiogram; EGM, intracardiac electrogram; RWPT, R-wave peak time; PVC, premature ventricular complex.

A 76-year-old woman with atrial fibrillation and third-degree atrioventricular block underwent LBBP. Echocardiography examination revealed left ventricular end-diastolic diameter of 44 mm and left ventricular ejection fraction of 75%. Uninterrupted ECG and EGM were recorded on an electrophysiology system using John Jiang’s connecting cable (Xinwell Medical Technology Co., Ltd., Ningbo, Zhejiang, China) (5, 6). In contrast to the interrupted pacing method, this continuous pacing and recording technique enables real-time monitoring of changes in ventricular COI and the paced QRS complex as the lead advances into the interventricular septum. We previously described the LBBP implantation procedure in detail (7, 8). During the entire screw-in process, the COI amplitude increased, peaked, and gradually decreased, followed by a small but significant abrupt decrease (Figure 1). Simultaneously, the impedance dropped from 691 to 532 Ω, and the myocardium capture was lost. This indicates septal perforation. No transition from left ventricular septal pacing to non-selective LBBP (NSLBBP) or NSLBBP to selective LBBP was observed during implantation. With no evidence of LBB capture, the electrode was retracted for the second implant. Repositioning to another site was uneventful. Smooth transition of paced QRS morphology from the LBB block pattern to the right bundle branch block pattern was observed as the lead advanced from the right to left side of the septum. After the COI amplitude peaked, the electrode was rotated very slowly to avoid a sudden drop in COI until the LBB area was reached. An abrupt shortening of the V6 R-wave peak time (V6RWPT) and a discrete EGM were subsequently observed (Figure 2), indicating LBB capture (9). The pacing threshold at the end of procedure was 0.7 V/0.5 ms. The lead was placed at a depth of 14 mm (Supplementary Figure).

## Discussion

Current of injury is a marker of active fixation electrode stability and the adequate capture threshold (10–12). COI, detectable on unfiltered EGM, is characterized by ST-segment elevation from baseline due to focal tissue trauma caused by the advancement of the lead tip into the septum. Su et al. described the COI abruptly disappeared when perforation happened (13). Ponnusamy et al. demonstrated that decreased COI amplitude differed significantly before versus after perforation (COI amplitude decreased from 15.4 ± 11.6 to 0.9 ± 0.6 mV) (3). However, the current intermittent pacing technique cannot monitor unfiltered EGM in real time. Therefore, it is unknown exactly when perforation occurs and how EGM changes therein. Some important practical considerations for implantation with deep septal lead deployment are when not to screw in further and when to reposition the lead.

Here we found a gradual increase in COI, followed by a gradual decrease, as the lead was gradually screwed into the septum. The possibility of microperforation should be considered after a small but significant, rather than dramatic, decrease in COI amplitude and the existence of impedance decrease with myocardial capture loss on unfiltered unipolar EGM (Figure 1, white arrow). Lead rotation should be stopped immediately to avoid complete entry into the ventricular cavity. The screw site must be repositioned, and the pacing output testing must be repeated in the same manner. The continuous pacing and recording technique in clinical practice allows real-time monitoring of the entire perforation process and early termination of the helix screwing to prevent immediate complete septal perforation in the event of a small and abrupt COI decrease.

Although changes in impedance and COI were observed during lead fixation, they were insufficient to confirm LBB capture. Because the myocardium and conduction system involve different tissues, they have different electrophysiological characteristics. Different ECG and EGM morphology was observed when different tissues were captured. Therefore, it is necessary to confirm LBB capture, demonstrated as dynamic changes in paced QRS morphology (14). However, subtle but significant changes in paced QRS morphology and EGM are difficult to observe in real time using the intermittent pacing technique but can be recorded by the continuous recording technique. When the rSR pattern suddenly changes to the r’SR pattern in lead V1 (Figure 2, blue rectangle) and V6RWPT abruptly shortens between the morphology of two adjacent paced QRS complexes (Figure 2, red arrow), the implantation process should be ceased, and threshold testing should be performed. When reducing the output, the discrete component in the filtered EGM and transition from the r’SR pattern to the M pattern on the ECG (Figure 2, green rectangle) are observed. In high- and low-output testing, V6RWPT is constant and remains the shortest value requiring measurement. If the V6RWPT of the high output is shorter than that of the low output, the lead must be screwed in slightly to keep the V6RWPT of the high and low outputs constant.

## Conclusion

First, when the COI amplitude peaks, the electrode must be rotated very slowly. Second, when the QRS morphology changes dynamically (such as V6RWPT abruptly shortening with the rSR pattern transition to the r’SR pattern) and there is evidence of LBB capture, the rotation should be stopped. Third, when a small but significant decrease in COI amplitude occurs with a decrease in impedance and loss of capture, the implantation should be stopped and the screw-in site be repositioned. This technique could be used to confirm LBB capture and avoid perforation.

## Data availability statement

The original contributions presented in the study are included in the article/Supplementary material, further inquiries can be directed to the corresponding author.

## Ethics statement

The studies involving human participants were reviewed and approved by Hwa Mei Hospital, University of Chinese Academy of Sciences Ethics Committee Review. The patients/participants provided their written informed consent to participate in this study and for the publication of this case report. Written informed consent was obtained from the individual(s) for the publication of any potentially identifiable images or data included in this article.

## Author contributions

All authors listed have made a substantial, direct, and intellectual contribution to the work, and approved it for publication.

## References

[1] Huang W Su L Wu S Xu L Xiao F Zhou X A novel pacing strategy with low and stable output: pacing the left bundle branch immediately beyond the conduction block. *Can J Cardiol.* (2017) 33:1736.e1–1736.e3. 10.1016/j.cjca.2017.09.013 29173611

[2] Huang W Chen X Su L Wu S Xia X Vijayaraman P. A beginner’s guide to permanent left bundle branch pacing. *Heart Rhythm.* (2019) 16:1791–6. 10.1016/j.hrthm.2019.06.016 31233818

[3] Ponnusamy SS Basil W Vijayaraman P. Electrophysiological characteristics of septal perforation during left bundle branch pacing. *Heart Rhythm.* (2022) 19:728–34. 10.1016/j.hrthm.2022.01.018 35066178

[4] Shali S Wu W Bai J Wang W Qin S Wang J Current of injury is an indicator of lead depth and performance during left bundle branch pacing lead implantation. *Heart Rhythm.* (2022) 19:1281–8. 10.1016/j.hrthm.2022.04.027 35500789

[5] Shen J Jiang L Cai X Wu H Pan L. Left bundle branch pacing guided by continuous pacing technique that can monitor electrocardiograms and electrograms in real time: a technical report. *Can J Cardiol.* (2022) 38:1315–7. 10.1016/j.cjca.2022.03.003 35276280

[6] Shen J Jiang L Wu H Cai X Zhuo S Pan L. A continuous pacing and recording technique for differentiating left bundle branch pacing from left ventricular septal pacing. *Can J Cardiol.* (2022) Epub ahead of print. 10.1016/j.cjca.2022.09.008 36113707

[7] Wu H Jiang L Shen J. Recording an isoelectric interval as an endpoint of left bundle branch pacing with continuous paced intracardiac electrogram monitoring. *Kardiol Pol.* (2022) 80:664–71. 10.33963/KP.a2022.0094 35380007

[8] Shen J Jiang L Jiang F Wu H Cai X Zhuo S Premature beat of selective left bundle branch: a novel marker for reaching and capturing the left bundle branch. *J Interv Card Electrophysiol.* (2022) Epub ahead of print. 10.1007/s10840-022-01203-2 35362830

[9] Wu H Jiang L Shen J Zhang L Zhong J Zhuo S. Electrophysiological characteristics and possible mechanism of bipolar pacing in left bundle branch pacing. *Heart Rhythm.* (2022) Epub ahead of print. 10.1016/j.hrthm.2022.06.022 35718314

[10] Saxonhouse SJ Conti JB Curtis AB. Current of injury predicts adequate active lead fixation in permanent pacemaker/defibrillation leads. *J Am Coll Cardiol.* (2005) 45:412–7. 10.1016/j.jacc.2004.10.045 15680721

[11] Redfearn DP Gula LJ Krahn AD Skanes AC Klein GJ Yee R. Current of injury predicts acute performance of catheter-delivered active fixation pacing leads. *Pacing Clin Electrophysiol.* (2007) 30:1438–44. 10.1111/j.1540-8159.2007.00889.x 18070296

[12] Shali S Su Y Qin S Ge J. Could persistency of current of injury forecast successful active-fixation pacing lead implantation? *Int J Cardiol.* (2018) 258:121–5. 10.1016/j.ijcard.2018.01.005 29428237

[13] Su L Xu T Cai M Xu L Vijayaraman P Sharma PS Electrophysiological characteristics and clinical values of left bundle branch current of injury in left bundle branch pacing. *J Cardiovasc Electrophysiol.* (2020) 31:834–42. 10.1111/jce.14377 32009260

[14] Jastrzębski M Burri H Kiełbasa G Curila K Moskal P Bednarek A The V6-V1 interpeak interval: a novel criterion for the diagnosis of left bundle branch capture. *Europace.* (2022) 24:40–7. 10.1093/europace/euab164 34255038 PMC8742628

