# Supplementary figures and images for: Case report: Left bundle branch pacing guided by real-time monitoring of current of injury and electrocardiography
Source: Front Cardiovasc Med. 2022 Nov 9;9:1025620. doi: 10.3389/fcvm.2022.1025620 (PMC9681806; doi:10.3389/fcvm.2022.1025620)

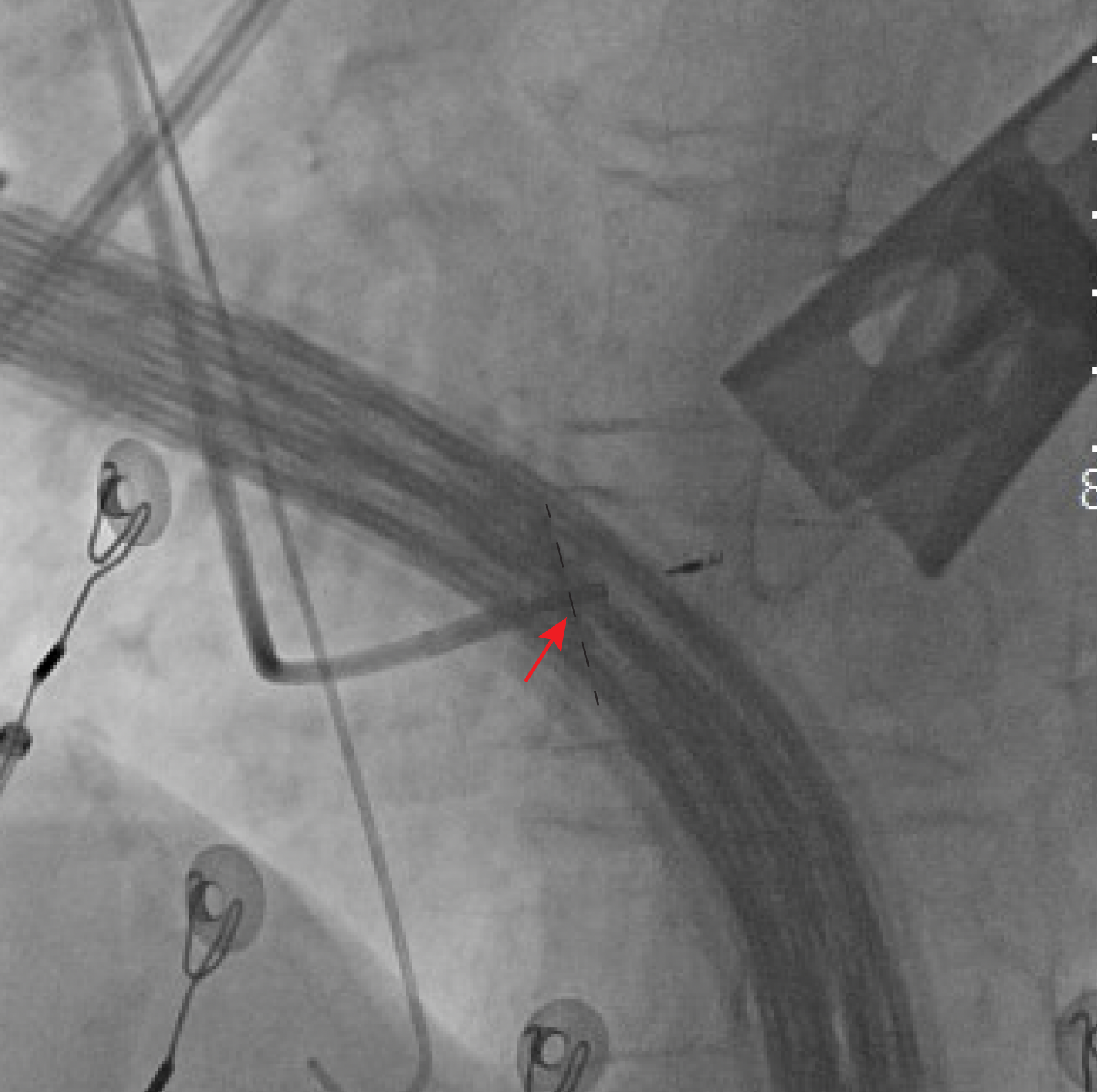

Supplement: Supplementary file 1 [file Data_Sheet_1.PDF]
